# The effects of high dose of two manganese supplements (organic and inorganic) on the rumen microbial ecosystem

**DOI:** 10.1371/journal.pone.0191158

**Published:** 2018-01-11

**Authors:** Svetlana Kišidayová, Peter Pristaš, Michaela Zimovčáková, Monika Blanár Wencelová, Lucia Homol'ová, Katarína Mihaliková, Klaudia Čobanová, Ľubomíra Grešáková, Zora Váradyová

**Affiliations:** 1 Institute of Animal Physiology, Slovak Academy of Sciences, Košice, Slovakia; 2 Institute of Biology and Ecology, University of P. J. Šafárik, Košice, Slovakia; University of Wisconsin Madison, UNITED STATES

## Abstract

Little is known about the effects of the high dose and types of manganese supplements on rumen environment at manganese intake level close above the limit of 150 mg/kg of dry feed matter. The effects of high dose of two manganese supplements (organic and inorganic) on rumen microbial ecosystem after four months of treatment of 18 lambs divided into three treatment groups were studied. We examined the enzyme activities (α-amylase, xylanase, and carboxymethyl cellulase), total and differential microscopic counts of rumen ciliates, total microscopic counts of bacteria, and fingerprinting pattern of the eubacterial and ciliates population analyzed by PCR-DGGE. Lambs were fed a basal diet with a basal Mn content (34.3 mg/kg dry matter; control) and supplemented either with inorganic manganous sulfate or organic Mn-chelate hydrate (daily 182.7, 184 mg/kg dry matter of feed, respectively). Basal diet, offered twice daily, consisted of ground barley and hay (268 and 732 g/kg dry matter per animal and day). The rumens of the lambs harbored ciliates of the genera of *Entodinium*, *Epidinium*, *Diplodinium*, *Eudiplodinium*, *Dasytricha*, and *Isotricha*. No significant differences between treatment groups were observed in the total ciliate number, the number of ciliates at the genus level, as well as the total number of bacteria. Organic Mn did decrease the species richness and diversity of the eubacterial population examined by PCR-DGGE. No effects of type of Mn supplement on the enzyme activities were observed. In comparison to the control, α-amylase specific activities were decreased and carboxymethyl-cellulase specific activities were increased by the Mn supplements. Xylanase activities were not influenced. In conclusion, our results suggested that the intake of tested inorganic and organic manganese supplements in excess may affect the specific groups of eubacteria. More studies on intake of Mn supplements at a level close to the limit can reveal if the changes in microbial population impact remarkably the other rumen enzymatic activities.

## Introduction

Nutritional requirements for Mn vary depending on the animal species and the stage of the life cycle. Ruminants are not well able to use Mn naturally present in the feed. Only 1% or less of manganese is absorbed from the ruminant diet [1]. Manganese is necessary component and cofactor of enzymes involved in lipid, carbohydrate and protein metabolism, fertility and immune functions [2–4], so Mn is added to various mineral food supplements. The maximum EU authorized total content of Mn is 150 mg/kg of complete feed for food-producing animals, except fish, for which it is 100 mg/kg [5]. Manganese oxide and manganese sulfate monohydrate are safe Mn sources for all animal species. The addition of these compounds at the permitted level does not threaten either the safety of consumers or the environment [5]. The organic forms of microelements are considered to be more available to animals [6,7]. Relative bioavailability of manganese from manganese methionine was 120% of that present in the sulfate form in experiments conducted with lambs [8]. Possible reasons may be that they form fewer complexes with other components of food, or they are less likely to cause adverse effects in the digestive tract [9]. The organic forms of Mn could have a more extensive use, and therefore, they can be added in smaller amounts to feed, since such Mn is more absorbed and is also less likely to be removed from the body, thus leading to less accumulation in the environment [10].

The impact of the high dose of Mn on rumen environment both in vivo and in vitro is little described. An increased amount of Mn in the diet could affect the activity of microorganisms or their produced enzymes. In the in vitro studies, the decrease of rumen cellulose digestion was observed with more than 100 μg/ml added inorganic Mn and the increase of rumen cellulose digestion was observed with 5–30 μg/ml added inorganic Mn [11,12]. Added inorganic Mn (100 ppm) increased in vitro dry matter digestibility in cattle rumen content fermentation in vitro [13]. The omission of Mn resulted in a significant lowering of the rumen cellulose digestion in vitro [14]. On the other hand, a high concentration of Mn can enhance the cellulolytic activities of other organisms, e.g., *Aspergillus niger*. The cellulase activities of *A*. *niger* were increased with added inorganic Mn in the range of 0.16 g/l to 0.59 g/l [15]. No other studies describe the effects of high Mn dose (either organic or inorganic) on rumen microbial activities or population composition.

This work contributes to the expansion of knowledge on the effects of Mn sources (organic and inorganic) on the rumen microbial ecosystem at Mn intake level close to the limit. We hypothesized that the dose of Mn close above the maximum authorized level (150 mg/kg) could affect the rumen microbial population composition and their activities irrespective of the Mn sources. In the present study, we investigated the effects of high dose of two manganese supplements (organic and inorganic) on some aspects of the lamb rumen microbial ecosystem. We examined the enzyme activities (α-amylase, xylanase, and carboxymethyl cellulase), total and differential microscopic counts of rumen ciliates, total microscopic counts of bacteria, and fingerprinting pattern of the eubacterial and ciliates population analyzed by polymerase chain reaction denaturing gradient gel electrophoresis (PCR-DGGE).

## Material and methods

The experimental protocol was approved by the Ethical Committee of the Institute of Animal Physiology, SAS, and the State Veterinary and Food Office (Ro-1479/11-221/3). All animals were kept, and the experimental procedures used in this study were performed in accordance with European Community guidelines (Directive 2010/63/EU).

### Animals, diets and experimental design

Eighteen ewe lambs of the Improved Valachian breed (wool type) with a mean age of 7 months and an initial average weight of 21.1±0.5 kg were used. Before experimentation, the lambs were reared with their mothers in one herd. The lambs were allotted in a completely randomized single factor design with three dietary treatments and six replicates. The animals were fed a basal diet providing 34.3 mg Mn/kg DM (Table 1). To examine the effects of high dose of two types Mn supplements the animals were treated with either inorganic manganese (IMn, 182.7 mg total Mn/kg DM) or organic manganese (OMn, 184 mg total Mn/kg DM). No manganese supplement was added to the control group. The three experimental diets were offered to lambs for a 16-week treatment period. Each lamb was fed a basal diet offered twice daily. The IMn as manganese sulfate monohydrate (MnSO_4_.H2O, Sigma-Aldrich, USA) or OMn as manganese glycine chelate (Glycinoplex-Mn22%, Phytobiotics, Futterzusatzstoffe GmbH, Eltville, Germany) was daily mixed with ground barley grains, and the barley consumption by each animal was visually checked. During the entire experimental period, the lambs were housed individually in 1.65×1.25 m pens with free access to fresh potable water through an automatic cup waterer. A trace mineral lick without Mn was offered to each lamb once a week (Table 1). Animals were slaughtered at the end of the experiment in an abattoir at the Institute of Animal Physiology (Košice, № SK U 07016). The lambs were not fed at the day of the slaughtering. Rumens were surgically removed, ligated at the reticulum and omasum, and transferred to the next lab for collecting samples of total contents. The content in isolated rumens was thoroughly mixed, and total rumen contents were collected for eubacterial and ciliate population PCR-DGGE analysis, for a microscopic count of ciliates and total bacteria and the evaluation of enzyme activities against polysaccharides (starch, xylan, and carboxymethyl-cellulose). For eubacterial and ciliate population DGGE analysis and evaluation of enzyme activities, the total rumen content of approximately 10 g/animal was divided into 2 ml Eppendorf microtubes and frozen at -70°C until analysis. For the microscopic counts, about 15 g/animal of total rumen content was preserved with an equal amount of 10% formaldehyde solution (w/w) in 50 ml polypropylene tubes with screw caps and stored at 8°C in a refrigerator until analysis.

**Table 1:** Composition of basal diet (g/kg DM).

Components	g/kg
Meadow hay	732
Ground barley	268
**Chemical composition**	
Dry matter	895
Crude protein	125
Acid detergent fiber	266
Neutral detergent fiber	396
Ash	66
Manganese	0.0343

Mineral lick composition without Mn (g/kg): Ca 16.2, Na 316, Mg 32, Cu 0.7, Zn 3.1, Co 0.06, I 0.02

### Qualitative analysis of the eubacterial and ciliated protozoa populations by PCR-DGGE

Total community DNA was isolated from rumen content samples (5 g) using a QIAamp DNA Stool Mini Kit (Qiagen, Valencia, CA, USA) according to the manufacturer's instructions. The quality of the community DNA was assessed by 0.8% agarose gel electrophoresis. DNA was visualized by ethidium bromide staining and recorded using the Gel Logic 212 PRO imaging system (Carestream, NY, USA). Total DNA (50 ng) was used as a template for PCR amplification of the 16S rRNA gene. In the first round of PCR, universal primers for 16S rRNA fD1 (5'-AGA GTT TGA TCC TGG CTC AG-3'), rP2 (5'-ACG GCT ACC TTG TTA CGA CTT-3') and conditions specified by [16] were used to amplify of about 1500 base pairs regions of the 16S rDNA genes. The obtained 16S rDNA fragments were subsequently used as a template for the second round of PCR using specific bacterial primers GC-clamp-968f (5'- CGC CCG GGG CGC GCC CCG GGC GGG GCG GGG GCA CGG GGG GAA CGC GAA GAA CCT TAC—3') and 1401r (5'- CGG TGT GTA CAA GACCC—3') and the conditions specified by [17]. For the analysis of protozoal populations approximately 200 bp of the 18S rDNA gene were amplified in one step using the primers (forward– 5-'GGT GGT GCA TGG CCG-3', reverse– 5'-AAT TGC AAA GAT CTA TCC C-3' with a 45 nucleotide GC-clamp linked to the 5‘ terminus of the reverse primer) and the conditions specified by [18]. All PCR reactions were performed in a 50 μL PCR mixture containing 1 μl of DNA, 1 x PCR buffer, 2 mmol/l MgCl2, 1 μl of a 200 μmol/l sample of each dNTP, 1.25 U Platinum Taq DNA polymerase (Invitrogen, CA USA) and 25 pmol of each primer using a MJ Mini thermal cycler (Bio-Rad Laboratories, USA). The specificity of PCR reactions was monitored by 1.2% agarose gel electrophoresis. PCR products generated with GC-968f and 1401r primers were subjected to DGGE analysis. DGGE was performed using the DCodeTM Universal Mutation Detection System (Bio-Rad Laboratories, Hercules, CA, USA). PCR reaction products in a total volume of 45 μl were loaded onto an 8% (w/v) polyacrylamide gel (40% Acrylamide-Bis 37.5:1) in 1 x TAE (40 mM Tris, 20 mM acetate, 1mM EDTA) containing a linear denaturing gradient ranging from 30–60% denaturant (100% denaturant solution consists of 7 M urea and 40% formamide). Electrophoresis was run for 17 h at a constant voltage of 50 V and a temperature 60°C. The ethidium bromide stained DGGE gels were recorded using the GelLogic Pro documentation system, and the DGGE fingerprints obtained were processed using Phoretix1D software (TotalLab Ltd, Newcastle upon Tyne, UK) without any user interference.

### Estimation of total bacterial and ciliated protozoal counts

The number of rumen ciliates was estimated by counting microscopically the protozoa in an aliquot of the suitably diluted sample streaked on a glass slide [19]. At least four replicates were counted per sample. The methyl green was used to reveal the ciliates nuclei. The iodine solution was used to reveal the skeletal plates. The abundance of total bacteria was estimated by direct bacterial count through image analysis of pictures taken under bright field illumination of dried smears of formaldehyde-fixed samples [20–22]. Two smears stained with methylene blue and with known dimensions and known volumes per sample (animal) were prepared according to the Breed method [23]. Twenty randomly selected pictures per smear were taken. The images were processed and analyzed using ImageJ software according to ImageJ software documentation [24]. Ciliates and bacteria numbers per gram of wet rumen content were expressed as geometric means of log-natural transformed values ± geometric standard deviation.

### Preparation of crude protein extracts and measurement of hydrolytic activities

Preparation of crude protein extracts from rumen contents, and measurements of hydrolytic enzyme activities against starch, xylan, and carboxymethyl-cellulose (CMC) were carried out according to [25]. Thawed samples of rumen contents were resuspended at a 1:5 ratio in citrate buffer [17] with pH 6.8 containing a protease inhibitor cocktail (Complete Mini EDTA-free protease inhibitor cocktail tablets, Roche Diagnostics, Meylan, France). The suspensions were then sonicated on ice (three times for 30 seconds, Ultrasonic Homogenizer 4710 Series; Cole-Parmer Instrument Co., Chicago, IL), and the sonicated extracts were used as crude protein extracts. The protein concentrations in the extracts were measured using the Bradford assay [26], with bovine serum albumin as the standard. The activities of polysaccharide-degrading enzymes were determined by measuring the amount of reducing sugar formation from the respective polymeric substrates after incubation with the crude protein extracts [27,28]. Hydrolytic reactions were performed at 39°C and pH 6.8 during 2h incubations. The hydrolytic activities were measured for each rumen content sample (animal) and each enzyme separately in three different assays (n = 6). Enzymatic activities were expressed in SI units, katals (1 kat = 1 mol/s). One katal is the amount of enzyme that converts 1 mole of substrate per second, so 1 U = 16.67 ŋkatal. Specific catalytic activities were expressed as microkatals of reducing sugar equivalent produced per gram of protein (μkat/g).

### Statistical analysis

Statistical analysis was performed using analysis of variance as a single factor design with three levels of treatment (Control, IMn, OMn). DGGE fingerprints were transformed into a band-matching table, and a dendrogram was constructed using the Dice similarity coefficient and UPGMA (Unweighted Pair Group Method with Arithmetic Mean) algorithm implemented in the Phoretix1D software package. Normalized band volumes calculated by Phoretix1D were used for calculation of diversity indices. The biodiversity indices (Shannon-Wiener diversity index, Evenness, and species richness) [29] were calculated for every lane using Species Diversity and Richness software version 4.1.2 (Pisces Conservation Ltd, Pennington, UK). Statistical differences in population variability were evaluated using One-way Anova with Bonferroni's Multiple Comparison Test. Ciliates and total bacteria microscopic counts were expressed per gram of wet weight of rumen content. The effects of manganese supplements on microbial counts (ciliates and bacteria), the Shannon-Wiener biodiversity index and genus evenness of major ciliates (*Entodinium*, *Eudiplodinium*, *Epidinium*, *Isotricha*, *Dasytricha*, and *Diplodinium*) were evaluated by the nonparametric Kruskal-Wallis method [30]. Differences in protein concentration and enzymatic activities were estimated by parametric One-way Anova with Bonferroni's Multiple Comparison Test. Only results obtained on rumen contents which were colonized by ciliates were statistically evaluated. Values of enzymes activities of rumen contents with missing out ciliates of organic Mn group were excluded from the statistical evaluation due to extreme values (n = 2). Treatment effects were determined to be significant at P < 0.05. GraphPad Prism software was used for statistical evaluations (GraphPad Software, Inc. San Diego, CA, USA).

## Results

### Qualitative analysis of eubacterial and ciliated protozoa populations by PCR-DGGE

**Fig 1:**
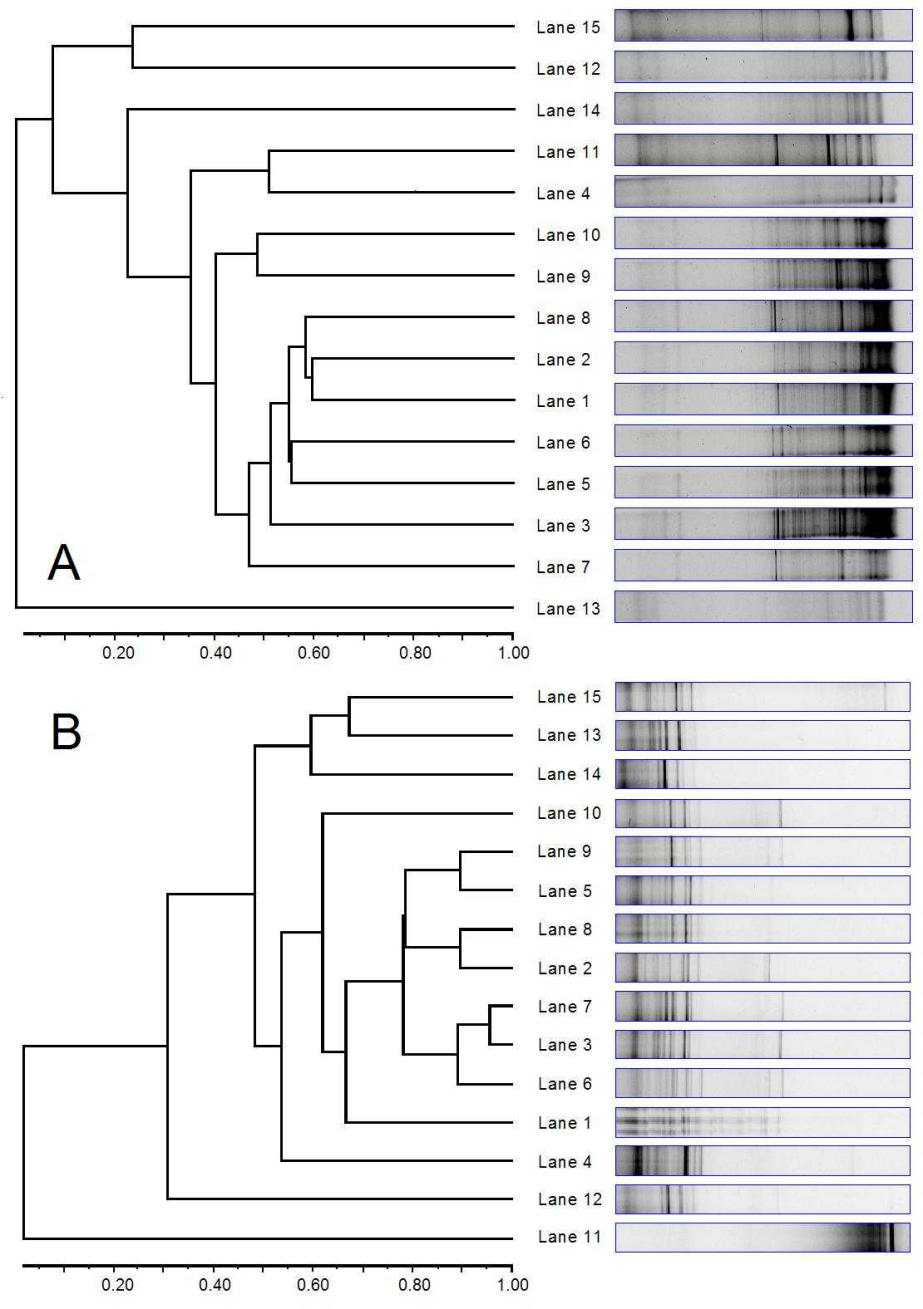
Effects of manganese on rumen microbial community. Principal component analysis of denaturing gradient gel electrophoresis (DGGE) patterns of the rumen eubacterial (A) and ciliated protozoal (B) community in lamb fed two types of manganese supplements for 16 weeks; Control group, Lanes 1–5; Inorganic Mn, Lanes 6–10; Organic Mn, Lanes 11–15.

DGGE analysis of the eubacterial population led to the detection of highly variable DGGE profiles showing from 7 to 28 bands per sample (Dice similarity coefficients 0.2–0.75). A similar dendrogram (Fig 1A) indicated that inorganic Mn did not influence the composition of the eubacterial population. Organic Mn addition, however, led to a shift in the eubacterial population. Samples from animals fed organic Mn showed the decreased variability of the eubacterial population, observed as a disappearance of bands. The average number of bands in samples from animals fed organic Mn was 11.6 compared to 20.2 and 22.8 for animals fed inorganic Mn or control animals, respectively. The Shannon-Wiener diversity index was statistically lower (P = 0.05) in animals fed organic Mn compared to animals fed inorganic Mn or control animals (1.8 compared to 2.5 for both control and inorganic Mn-supplemented animals). DGGE analysis of the population of ciliated protozoa led to the detection of 5 to 16 bands per sample. Similar banding patterns were observed for most samples (Dice similarity coefficients 0.6–0.9), except sample 11. The similarity dendrogram (Fig 1B) indicated that Mn supplements did not influence the composition of the protozoal population. The average Shannon-Wiener diversity index of the protozoal population of animals fed organic Mn was 1.5 compared to 1.9 and 1.8 for animals fed inorganic Mn or control animals, respectively. The differences, however, were not statistically significant (Table 2, P > 0.05).

**Table 2:** Evaluation of population variability of DGGE profiles and microscopic counts of lamb rumen eubacteria and ciliates with two manganese supplements.

Item	Eubacteria (DGGE)			Ciliates (DGGE)			Ciliates genera*(microscopic)		
	Control	Inorganic Mn	Organic Mn	Control	Inorganic Mn	Organic Mn	Control	Inorganic Mn	Organic Mn
Species richness	22.8 ± 5.26 ^b^	20.2 ± 3.35 ^b^	11.2 ± 4.15 ^a^	9.8 ± 3.49	8.8 ± 1.30	7.6 ± 1.67	4.7 ± 1.03	4.8 ± 0.98	3.2 ± 2.56
Diversity (Shannon index)	2.5 ± 0.33 ^b^	2.5 ± 0.22 ^b^	1.8 ± 0.39 ^a^	1.8 ± 0.32	1.9 ± 0.26	1.5 ± 0.27	0.8 ± 0.33	0.6 ± 0.25	0.4 ± 0.31
Evenness (Pielou index)	0.81 ± 0.067	0.82 ± 0.029	0.74 ± 0.091	0.78 ± 0.068	0.82 ± 0.147	0.77 ± 0.071	0.78 ± 0.068	0.39 ± 0.147	0.26 ± 0.199

Within a row, means with a different superscript letter (a, b) differ at P < 0.05

* Microscopic counts of major genera of ciliates (*Entodinium*, *Eudiplodinium*, *Epidinium*, *Isotricha*, *Dasytricha*, and *Diplodinium*)

Number of bands were evaluated in DGGE profiles; Values are means ± standard deviation

### Effects of manganese supplements on total bacterial and ciliated protozoal counts

Rumens of lambs were colonized with the B type population of ciliated protists, except two animals in the group with OMn supplement (Table 3). Major genera were *Entodinium* (76–84%), *Epidinium* (*E*. *ecaudatum caudatum*, 8–13%), *Eudiplodinium* (*E*. *maggii*, 2–4%), *Dasytricha* (*D*. *ruminantium*, 5–9%) and *Isotricha* (*I*. *intestinalis*, *I*. *prostoma*, 1–2%). Few empty pellicles of large ciliates and a few disintegrated small ciliates were visible microscopically in the rumen contents of two animals of the group with OMn supplement (S1 Fig). Average total microscopic counts of ciliates were 13.13–13.68 per gram of wet rumen content, expressed as geometric means of log-natural transformed values. Average total microscopic counts of prokaryotes were 23.26–23.60 per gram of wet rumen content, expressed as geometric means of log-natural transformed values. No effects of type of manganese supplements on the microbe counts were observed at the tested doses (Table 3). No treatment effects were observed on the population variability of the major genera of ciliates (*Entodinium*, *Eudiplodinium*, *Epidinium*, *Isotricha*, *Dasytricha*, and *Diplodinium*), expressed as the Shannon-Wiener diversity index and Evenness index (Table 2).

**Table 3:** Effects of elevated intake of two manganese supplements (inorganic, IMn, and organic, OMn) on rumen microbial populations and enzyme activities.

	Control	IMn	OMn	P value	OMn, no ciliates
Mn daily intake (mg/head)	34	181	182	-	182
Animal counts	6	6	4	-	2
Total ciliate count	13.13 ± 1.678	13.68 ± 1.460	13.48 ± 1.508	0.34	ND
*Entodinium*	12.84 ± 1.734	13.47 ± 1.558	13.31 ± 1.513	0.17	ND
(%)	76 ± 13.0	81 ± 7.5	84 ± 1.6		
*Epidinium*	11.09 ± 1.930	11.31 ± 1.710	10.98 ± 1.781	0.81	ND
(%)	13 ± 7.2	11 ± 4.3	8 ± 1.4		
*Eudiplodinium*	9.92 ± 1.352	9.66 ± 2.404	9.57 ± 1.128	0.58	ND
(%)	4 ± 2.0	3 ± 2.2	2 ± 1.1		
*Isotricha*	7.75 ± 6.693	8.95 ± 1.355	8.27 ± 1.524	0.35	ND
(%)	2 ± 1.3	1 ± 0.6	1 ± 0.2		
*Dasytricha*	10.60 ± 1.707	10.77 ± 1.281	10.53 ± 1.388	0.69	ND
(%)	9 ± 4.0	6 ± 2.4	5 ± 0.9		
Total bacteria count	23.51 ± 1.513	23.60 ± 1.223	23.32 ± 1.238	0.1	23.26 ± 1.299
Protein conc. (g/kg)	1.8 ± 0.46	2.1 ± 0.38	2.1 ± 0.77	0.43	0.4 ± 0.01
**α-amylase**					
Katal. activity	4.5 ± 2.23	3.4 ± 0.83	3.9 ± 0.89	0.57	2.9 ± 1.51
Spec. katal. activity	3.3 ± 0.58	2.1 ± 0.66 *	2.4 ± 0.20	0.02	25.3 ± 8.41
**CMC-ase**					
Katal. activity	2.1 ± 0.04	1.8 ± 0.86	1.8 ± 0.66	0.77	0.9 ± 0.60
Spec. katal. activity	0.6 ± 0.26	1.5 ± 0.35 *	1.6 ± 0.26 **	0.004	4.8 ± 3.70
**Xylanase**					
Katal. activity	149.8 ± 27.0	154.3 ± 31.20	147.6 ± 33.96	0.96	22.2 ± 22.25
Spec. katal. activity	154.6 ± 13.71	196.6 ± 48.24	212.4 ± 59.50	0.71	90.9 ± 53.74

Ciliates (bacteria) numbers per gram of wet rumen content were expressed as geometric means of log-natural transformed values ± geometric standard deviation; %, genus percentage of total ciliates number; ND, not detectable; CMC-ase, carboxymethyl-cellulase; Counts of microbes, protein and enzymes values are means ± standard deviation; Katalytic activity, μkat/l; Specific katalytic activity, μkat/g protein. Within a row, means with a superscript symbol (*) differ against to control group

* P < 0.05

** P < 0.01.

### Effects of manganese supplements on protein content and polysaccharide hydrolytic activities

No effects of type of manganese supplements were observed on protein concentration in rumens harboring ciliates (Table 3). The protein concentrations were low in two rumens without observable live ciliates (supplemented with organic manganese). Alfa-amylase specific catalytic activities were decreased in rumen contents supplemented with inorganic manganese in comparison to the control group (P < 0.021). CMC-ase specific katalytic activities were increased in both supplemented groups in comparison to the control group (IMn, P < 0.05; OMn, P < 0.01). Manganese supplements did not influence xylanase activity.

## Discussion

In general, the count of ciliated protists in rumens varies from a few thousand to millions of protozoa per gram of rumen content, depending mainly on the feed taken by the host [19,31]. In our samples, we estimated the number of ciliates microscopically from 56.3 × 10^4^ to 93.3 × 10^4^ per gram of wet rumen content (13.13–13.48 as log-natural transformed values). The numbers of rumen bacteria occur in the range of 10^9^–10^11^ per gram of wet rumen contents [31,32]. In our samples, we estimated the number of bacteria from 1.35 × 10^10^ to 1.81 × 10^10^ per gram of wet rumen contents (23.26–23.60 as log-natural transformed values). Their counts were in the range typical for domesticated ruminants. PCR-DGGE detects only a limited part of the microbial diversity in a rumen because the diversity of bacteria in the rumen is extraordinarily high. Rumen bacterial community may easily contain more than 10,000 different species, while the DGGE resolution of more than 20–50 bands on a gel is difficult [33]. DGGE can evaluate just the most dominant members of the bacterial community—accounting for at least 3% of total population. DGGE profiles demonstrate the overall changes in community documented by the composition of abundant members of the community. DGGE seems to be useful for assessing changes in the main constituents of rumen microbial community. In our study, DGGE analysis revealed a decrease of eubacterial variability in the group supplemented with organic manganese. Similar effects of inorganic manganese of 0.013–0.045 g/kg of feed dry matter on sheep rumen prokaryotes were observed in the study of [34], where manganese treatments did not affect the total number of rumen bacteria or the size structure of bacteria population. Our results point to the potentially adverse effects of the high experimental dose of organic manganese on some groups of rumen microbes. It seems that organic manganese may be more accessible (bioavailable) for rumen microbes, resulting in their higher sensitivity in comparison with a similar concentration of inorganic manganese. The analysis of Mn content in whole cells of rumen microbes could validate the assumption on the better bioavailability of organic Mn.

We cannot explain the absence of live ciliates in the rumen contents of two lambs by the action of the organic manganese supplement exclusively because we did not examine the animals before experimentation. On the other hand, the spontaneous disappearance of protozoa from rumens can occur in young lambs [35]. However, the presence of empty pellicles and disintegrated ciliates points to recent events of ciliates death. According to authors’ long-term experiences, the presence of empty pellicles in rumen content point to the death of ciliates approximately 24-48h ago. In rumen content, the pellicles of ciliates persist no longer than 48h. Usually, pellicles are not present in rumen contents at the presence of live protozoa. Otherwise, that means, that ciliates colonized rumens. However, these indices did not correspond with the DGGE analysis, which revealed ciliates bands in the respective samples (see L11, L15). It seems the DNA of ciliates is relatively resistant to gut microbial digestion. They can be yet detected in feces [36] though no live ciliates have been observed in the feces of ruminants to date, except parasitic *Buxtonella* species [37]. Therefore, interpretation of molecular data should be made with caution, if no other observations (e.g., microscopic) are made.

Rumen protozoa may impact the level of microbial protein synthesis since they prey on rumen bacteria [19,38–40]. Several studies revealed that the absence of ciliated protists resulted in a higher number of bacteria and higher synthesis of microbial protein in the rumen [41–43]. However, we did not record any differences in the numbers of bacteria in the rumen samples of two ciliates-free animals compared to faunated animals. Also, the amount of protein in these samples was much lower than in the others, which does not coincide with previous studies. The type of analysis can cause contradictions in the concentration of proteins in our results and the results of other works. On the other hand, a more probable reason may be that there was not enough time to compensate microbial protein by the growth of prokaryotes after the probably recent event of ciliates death. However, we observed a slight nonsignificant increase of protein contents of Mn treated groups which may impact some enzyme activities.

Rumen ciliates can remarkably influence hydrolytic activities in the rumen. The effect of manganese on the enzymatic hydrolytic activities of rumen microorganisms is poorly studied. In our study, we observed a decrease the amylolytic specific catalytic activity only in the group with inorganic Mn in comparison to the control group. The results are consistent with other studies in which a negative impact of ciliates on rumen amylolytic bacteria was found [44,45]. CMC-ase specific activity was increased by both Mn supplements in comparison to control group. Manganese is an important lignocellulolytic agent [15]. Hydrolysis of cellulose in an *in vitro* rumen mixed culture was affected by Mn in the study of [15]. The omission of Mn significantly reduced cellulolytic activity *in vitro* in the study of [14]. On the other hand, a high dose of Mn resulted in the inhibition of cellulolytic activity *in vitro* [46]. Rumen ciliates can contribute significantly to the fiber digestion in the rumen. The presence of ciliates can stimulate cellulolysis by the bacteria. Studies in vitro and in vivo indicated that approximately a quarter to one-third of fiber breakdown in the rumen was protozoal [47,48]. High dose of either organic or inorganic Mn has no effects on xylanase activity. We found no effects of type of Mn supplements on the measured enzyme activities, in contrast to studies which claim that organic forms of Mn are biologically more accessible and more usable [9,49,50].

## Conclusion

The results of this work suggest that the high dose of Mn close to the limit, both inorganic and organic, does not affect the number and species composition of ciliates and the number of bacteria. However, it may influence the composition of the eubacterial community and the enzymatic activities of rumen microorganisms, especially amylolytic and cellulolytic activities.

## Supporting information

S1 Fig The image of rumen content with the pellicles of dead rumen protozoa. The red arrows point to the pellicles of dead rumen ciliates of un lamb of the OMn group (scale bars indicate 10 μm).
(TIF)
