# Supplementary figures and images for: The effects of high dose of two manganese supplements (organic and inorganic) on the rumen microbial ecosystem
Source: PLoS One. 2018 Jan 11;13(1):e0191158. doi: 10.1371/journal.pone.0191158 (PMC5764370; doi:10.1371/journal.pone.0191158)

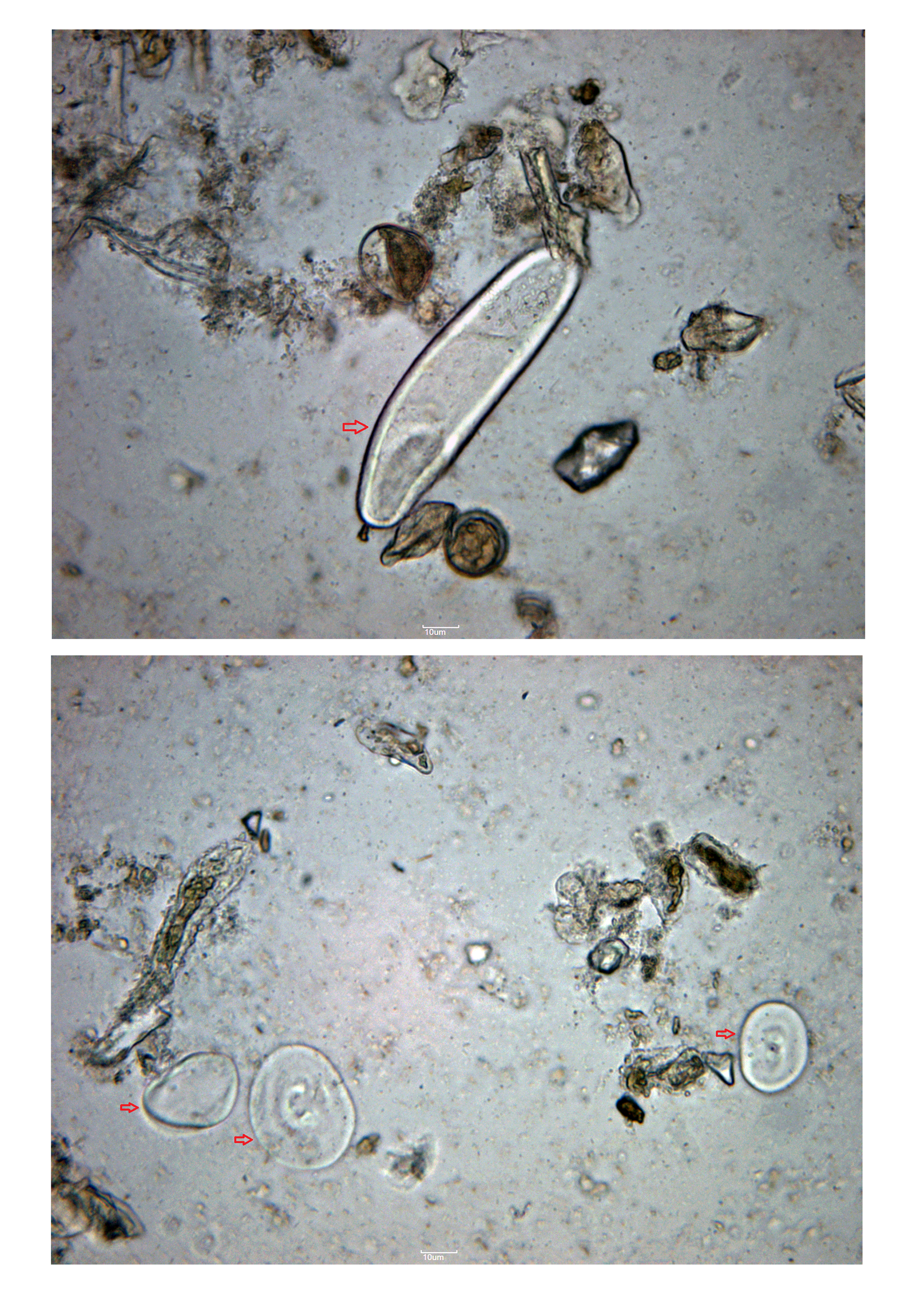

Supplement: S1 Fig — The red arrows point to the pellicles of dead rumen ciliates of un lamb of the OMn group (scale bars indicate 10 μm). (TIF) [file pone.0191158.s001.tif]
